# Animal models and experimental medicine and the Nobel Prize in Physiology or Medicine 2021—TRPV and PIEZO receptors for temperature and touch sensation

**DOI:** 10.1002/ame2.12196

**Published:** 2021-12-07

**Authors:** Yu Zhang, Dongyuan Zhang, Chuan Qin

**Affiliations:** ^1^ Institute of Laboratory Animal Sciences Chinese Academy of Medical Sciences Beijing China; ^2^ Comparative Medicine Center Peking Union Medical College Beijing China; ^3^ NHC Key Laboratory of Human Disease Comparative Medicine Beijing China; ^4^ Beijing Engineering Research Center for Experimental Animal Models of Human Critical Diseases Beijing China; ^5^ Chinese Association for Laboratory Animal Sciences Beijing China

## 1 INTRODUCTION

For decades, experimental animal models have been powerful tools for biomedical research and have supported most of the physiological or medical achievements recognized by Nobel Prizes, including the research that won this year's Physiology or Medicine Prize. On 4th October 2021, the Nobel Prize in Physiology or Medicine 2021 was awarded jointly to David Julius and Ardem Patapoutian "for their discoveries of receptors for temperature and touch."^1^ Their discoveries have profoundly changed our view of how we sense the world around us.^2^

The senses of temperature and touch are among the most basic physiological functions in animals and humans. Stimuli from the surrounding environment or physical objects interact with receptors, mainly temperature and tactile sensory receptors, and are converted into information, or neural signals. Subsequently, all the encoded neural signals are transmitted through afferent (or sensory) neural fibers to interneurons or the central nervous system, and thence through efferent neurons (or motor neurons) to effector organs, for example muscles. These steps form a complete reflex arc comprising the body's response to external stimuli. Perceptions of the external stimuli help our bodies to react, to make decisions and motor responses, so that we can feel, interpret and interact with our external and internal environment.

In mechanistic research, laboratory animal experiments focus on several major questions: What are the molecular bases to sensing heat, cold or mechanical force? How do external stimuli convert into electrical nerve signals? The winners of this year's Medical Prize investigated and identified the receptors for temperature and touch sensation. Their research used different species of laboratory animals and different strains of rodent animal models to unravel these questions.

## 2 ANIMAL MODELS FOR RESEARCHING TEMPERATURE‐SENSING MECHANISMS

For centuries, people have sought the secret of temperature perception. Since ancient times in China, the theory of cold/hot properties has been the most basic aspect of Traditional Chinese Medicine (TCM).^3^ The diagnosis of diseases differs according to their cold or hot properties and the treatment of the diseases takes advantage of the cold or hot medical properties of Chinese herbal medicines (CHMs).^4^ Besides non‐herbal medicines, acupuncture and moxibustion are among the most basic therapies of TCM. During the treatment, heat‐sensitization responses are the essential therapeutic effects.^5^, ^6^

In western countries, exploration of temperature sensation dates back to the 17th century, when the famous French philosopher René Descartes postulated that heat could send signals to the brain, in his book *Treatise of Man* (or *L'homme* in French). Centuries later, the 1944 Nobel Prize in Physiology or Medicine was awarded jointly to Joseph Erlanger and Herbert Spencer Gasser "for their discoveries relating to the highly differentiated functions of single nerve fibres."^7^ They divided nerve fibres into specific types with different transmission speeds for electrical nerve signals.^8^ Their discoveries demonstrated the ‘highway’ for electrical nerve signals to the central nervous system. However, how heat converts into electrical nerve signals remained unknown within this theoretical system.

The story of finding the molecular bases for sensing heat or cold began with chili peppers. When eating (or even touching) spicy chili peppers, the active component, capsaisin (8‐methyl‐*N*‐vanillyl‐6‐nonenamide), can induce a ‘hot’ and burning sensation in people. In the late 1990s, Dr Julius and colleagues tried to isolate a functional cDNA encoding a capsaicin receptor in sensory neurons. In 1997, they shared their discovery of a non‐selective cation channel TRPV1 (transient receptor potential vanilloid type 1, or VR1 for short), which can also be activated by noxious temperature stimuli. Using laboratory rats for their experiments, they found that VR1 is expressed in adult rats in primary sensory neurons, in a subset of sensory ganglion cells, including dorsal root ganglia (DRG) and trigeminal ganglia (TG) mainly in smaller‐diameter cell bodies instead of large‐diameter ones.^9^ TRPV1 is also expressed in spinal cord laminae I and II, solitary tract and area postrema of the caudal medulla.^10^ To further decode the TRPV1 receptor, transgenic mice models lacking the capsaicin receptor were developed. VR1^−/−^ mice showed little thermal hyperalgesia behavior, while the mechanical pain remained. This indicated that the TRPV1 receptor is essential to thermal hyperalgesia sensation.^11^

Thereafter, another chemical, a cooling agent, menthol, was used to identify the receptor for cold sensation. In 2002, Patapoutian and colleagues described the characterization of TRPM8, which can be activated both by menthol and cold temperatures.^12^, ^13^ The ion channels TRPV1 and TRPM8 belong to the family of transient receptor potential (TRP) ion channels. Later, other related receptors for more delicate thermal sensation, the TRP family, were identified,^14^ and these molecular receptors for temperature sensation were subsequently decoded.

Rodent models are useful for temperature research and have helped to provide a comprehensive understanding of temperature sensation. Using genetics and in vivo calcium imaging, researchers found that heat and cold could send major inputs to TRPV1^+^ or TRPM8^+^ DGR neurons and induce robust calcium responses in spinal neurons.^15^ Research into the properties of TRP receptor families, including their physiological and pathological functions in thermal sensation, chronic inflammatory pain and cancer pain, continues to take place.^16^, ^17^, ^18^, ^19^, ^20^, ^21^, ^22^

## 3 ANIMAL MODELS FOR RESEARCHING TOUCH PERCEPTION

The basic question of touch and pressure sensation is similar to that of the molecular mechanisms of temperature sensation: how do the external signals convert into electrical neural signals?

Originally, Ardem Patapoutian and colleagues identified a mechanosensitive cell line, Neuro2A cell. By poking at the cells with a micropipette, they analyzed 72 candidate genes and finally identified an unknown mechanically activated cation channel, which was later named PIEZO1, named after the Greek word for pressure “piesi”. PIEZO2 was later identified. Both PIEZO1 and PIEZO2 have apparent pressure sensing properties.^23^ Through rodent experiments, they found that PIEZO2 was expressed in different types of sensory neurons of DRG and in cutaneous mechanoreceptors known as Merkel cells. In 2014, a PIEZO2^CKO^ mice model was developed. Mice without PIEZO2 in both adult sensory neurons and Merkel cells had a profound deficit of touch sensation, even light touch, but other somatosensory functions maintain normal.^24^ Subsequent research has shown that the physiological functions of these PIEZOs include pressure sensating properties for touch, pain, proprioception, and even blood pressure regulation and lung inflation.^25^

## 4 CONCLUSION

The Noble Prizes were set up in 1895 by the Swedish chemist Alfred Bernhard Nobel (1833–1896), and the first awards of Physics, Chemistry, Peace and Literature Prizes were made in 1901. Since then, there have been 112 Nobel Prizes in Physiology or Medicine and 224 Nobel Prize laureates, most of whom relied on animal models in their experimental medical research. With further developments in science technology and increasingly open platforms for science sharing, experimental medicine based on laboratory animal resources will generate even more impetus for better research, better science and a better world.

## CONFLICT OF INTEREST

The authors have declared no conflicts of interest.

## AUTHOR CONTRIBUTIONS

Yu Zhang designed the project and wrote the manuscript; Dongyuan Zhang collected the references and wrote the manuscript; Chuan Qin designed the project and approved the final version.
